# Composition, antibiotic resistance, and virulence analysis of microbiota in dormitory drain pipes

**DOI:** 10.3389/fmicb.2023.1272605

**Published:** 2023-11-13

**Authors:** Yan Hu, Kunyuan Zhang, Nan Li, Shengqin Wang

**Affiliations:** ^1^National and Local Joint Engineering Research Center of Ecological Treatment Technology for Urban Water Pollution, Wenzhou University, Wenzhou, China; ^2^Zhejiang Provincial Key Laboratory for Subtropical Water Environment and Marine Biological Resources Protection, Wenzhou University, Wenzhou, China; ^3^College of Life and Environmental Science, Wenzhou University, Wenzhou, China

**Keywords:** microbiota, metagenomics, antibiotic resistance, virulence, drain pipes

## Abstract

**Introduction:**

Dormitory washbasins can breed microorganisms that produce odorous gases, polluting the indoor environment.

**Methods:**

We utilized metagenome sequencing to analyze the microbiota of 40 samples from the drain pipes of dormitory washbasins. Our study aimed to investigate the microbial community structure, antibiotic resistance genes, and virulence factors, and to identify potential influencing factors such as gender, hometown, frequency of hand sanitizer usage, and number of dormitory residents.

**Results:**

The analysis revealed 12 phyla and 147 genera, with *Proteobacteria* and *Actinobacteria* being the dominant phyla, and *Mycobacterium* and *Nakamurella* being the dominant genera. We found that the factors influencing the microbial community structure of the dormitory washbasin drain pipe are complex. The investigated factors have a slight influence on the drain pipe microbial community, with gender exerting a discernible influence. The annotation results revealed the presence of various virulence factors, pathogenic toxins and antibiotic resistance genes, including 246 different toxin types and 30 different types of antibiotic resistance genes. In contrast to the observed differences in microbial composition among samples, the distribution of resistance genes shows relatively small changes among samples. Antibiotics should be a contributing factor in the overall increase of antibiotic resistance genes in drain pipes.

**Discussion:**

Overall, our study provides important insights into the community structure and function of microorganisms in dormitory drainage systems, and can guide efforts to prevent and control microbial pollution.

## 1. Introduction

Dormitory washbasins are primarily used to discharge all kinds of washing water, which can result in scaling on the inner wall of pipes. The humid and dark environment inside of the pipes provides an ideal breeding ground for various microorganisms, which produce odorous gases through metabolism and can pollute the indoor environment. For example, the sink drain is a niche for *Mycobacterium*, among which many types of non-tuberculous mycobacteria are often considered environmental pathogens that can cause a range of human diseases (Ichijo et al., 2014; Gebert et al., 2018). Several studies have shown that pathogenic microorganisms can be transmitted and spread through the air or aerosols, infiltrating the human respiratory system and having significant impacts on various aspects of human society, including health and the economy (Darquenne, 2012). For instance, research has indicated that toilets can easily create negative pressure, leading to the spread of microorganisms into indoor spaces through aerosolization (Lou et al., 2021). This is particularly relevant in the context of the ongoing COVID-19 pandemic, where aerosols have emerged as a recognized significant transmission route. Consequently, the dissemination and propagation of microorganisms from drain pipes through airborne aerosols may lead to persistent indoor infections and disease transmission. Therefore, it is imperative to conduct further examination of their composition and influencing factors in order to advance our understanding.

Several factors can potentially influence the microbial community within drain pipes. It is evident that the oral flora of individuals plays a significant role in shaping the microbial composition of washbasin drain pipes, primarily due to daily oral hygiene practices. Studies have shown that human oral flora differs between genders (Galvão-Moreira et al., 2018), which may be related to differences in living habits (Stahringer et al., 2012). Students from different geographical regions have varying living habits, natural environments, social environments, and medical and health conditions. For example, the fluoride content in drinking water in different regions can greatly influence the structure of human oral flora (Dagli et al., 2016). The number of dormitory residents also impacts the microbial sources, such as oral flora and human sebaceous gland secretions, that affect the structure of the drain pipe flora. Moreover, the use of hand sanitizer can effectively reduce microorganisms on the skin surface (Jing et al., 2020), thereby directly or indirectly impacting the microbial community composition of drain pipes in dormitories.

Studying antimicrobial resistance genes (ARGs) and virulence factors of relevant pathogens in drain pipes is crucial for assessing the pathogenic potential of bacteria and establishing a theoretical foundation for microbial risk assessment. This is particularly important considering the emergence of ARGs and drug-resistant bacteria due to the widespread use of antibiotics and the significant research focus on environmental pollution of antibiotics. Biofilm adhering to the internal surface of pipes promotes high resistance of microorganisms to antibacterial drugs and creates favorable conditions for conditional pathogens (Rather et al., 2021). Human activities can also affect bacterial evolution, potentially leading to the emergence of new drug-resistant genes (Larsson and Flach, 2022). These drug-resistant genes can further spread through horizontal gene transfer mechanisms, accelerating the development of drug resistance (Hawkey and Jones, 2009). Virulence factors, including toxins, play a key role in the pathogenesis of bacterial infections. Pathogens invade hosts and multiply with the coordination of virulence factors, which can directly or indirectly cause disease.

Presently, our understanding of the composition and functionality of pathogenic microorganisms in the drain pipes of dormitory washbasins is limited. Here, metagenomic sequencing technology was utilized to identify the microbial community present in sediment samples collected from the drain pipes of dormitory washbasins. Subsequently, we investigated the impact of external factors, such as gender, place of hometown, frequency of hand sanitizer usage, number of dormitory residents, and occurrence of cold and antibiotic medication use, on the microbial community in the drain pipes. The microbial community composition, ARGs, and virulence factors were also analyzed, with a focus on identifying microbial characteristics that impact human health. This research is important to understand the community structure and function of microorganisms in dormitory drain pipes, to better prevent and control microbial pollution, and to ensure the health and safety of residents. Furthermore, it provides new ideas and directions for developing technologies and strategies to mitigate microbial pollution.

## 2. Materials and methods

### 2.1. Sample collection

To collect data on relevant influencing factors, such as gender, number of dormitory residents, and hometowns, 40 dormitories were selected as sampling subjects and conducted a questionnaire survey to determine the frequency of hand sanitizer usage, the occurrence of cold, and the antibiotic drugs usage. The age range of dormitory members is approximately 20 years old. To sample the inner wall of the drain pipe, we used two cotton swabs, each 15 cm in length, to evenly smear the pipe wall clockwise for 20 times at the nozzle 4–5 cm. We adjusted the position of the cotton swabs both inside and outside when smearing for the 11th time to ensure full contact with the collected samples. After removing the handle part with sterilized scissors, we placed the remaining cotton swab into a centrifuge tube coated with tin foil paper and stored it in the refrigerator at −80°C.

### 2.2. DNA extraction, library preparation, and sequencing

The samples consisted of 20 from male dormitories, 20 from female dormitories, and an additional two duplicate samples were included. These samples were frozen using dry ice and sent to Novogene Corporation in Beijing, China for microbial DNA extraction, employing a TIANGEN kit as per the manufacturer’s instructions. The DNA purity and integrity were assessed through agarose gel electrophoresis (AGE), while the DNA concentration was precisely determined using the Qubit 2.0 system. To construct the library, high-quality DNA samples were randomly fragmented into approximately 350 bp fragments using a Covaris ultrasonic crusher. These fragments were then subjected to end-repair, A-tailing, and ligation with adapters. Following library preparation, the Qubit 2.0 system was employed to initially quantify the library, which was subsequently diluted to a concentration of 2 ng/μL. The insert sizes of the library were evaluated using an Agilent 2,100 instrument to ensure they met the desired specifications. To ensure the library’s quality, real-time q-PCR was utilized to accurately determine the effective concentration of the library, which needed to exceed 3 nM. Once the library passed the quality inspection, sequencing was carried out on an Illumina HiSeq sequencing platform.

### 2.3. Quality control, annotation, and comparation

We conducted quality control and filtering on the original sequencing data using KneadData^1^, removing any sequences related to the human genome. Using MetaPhlAn 3.0, we annotated the sample data and obtained information on the relative abundance of microbial species at various taxonomic levels. MetaPhlAn 3 estimates the coverage of each marker and calculates the coverage of a clade by taking the robust average of the coverage values across all markers belonging to that clade. To obtain the relative abundance of each taxon, the clade coverages are then normalized across all detected clades (Truong et al., 2015). Both Bray–Curtis dissimilarity and Euclidean distance were employed for nonmetric multidimensional scaling (NMDS) analysis (*k* = 2) to evaluate the taxonomic similarity of microbial community in dormitory drain pipes. The significance of influencing factors on the NMDS ordinations was assessed using the envfit functions from the R package vegan (v2.6-4). Anosim analysis was performed using R software to determine whether the differences between groups were significantly different from those within groups. We calculated the alpha diversity indices of the sample microbiota, including the Shannon, Simpson, and Chao1 indices, using QIIME 2 to compare the diversity among the microbiota samples (Bolyen et al., 2019). We used the ordinal logistic regression analysis with SPSS 23.0 to evaluate whether there were significant differences in alpha diversity indices among the various groups. Using the LEfSe analysis platform (Segata et al., 2011), we conducted a comparative analysis between groups and subgroups within groups to identify species with significant differences in abundance.

### 2.4. Prediction and analysis of drug resistance genes and virulence factors

PathoFact (v1.0) is a highly accurate tool that combines various existing modules and databases to create a pipeline for identifying virulence factors, bacterial toxins, and ARGs from metagenomic data in a contextualized manner (de Nies et al., 2021). We employed PathoFact to compare with CARD and ARDB, respectively, for the annotation of ARGs and investigation of drug resistance in the samples. Additionally, we utilized this tool to annotate virulence factors in comparison to the VFDB database. The predicted protein sequences of ARG ORFs were annotated using DIAMOND against non-redundant protein NCBI databases with an *E*-value threshold of ≤1 × 10^−5^. Subsequently, the results were further annotated using MEGAN (MEtaGenome ANalyzer, Version 6) to assign taxonomic genera to the sequences (Huson et al., 2011).

The metagenomic reads were aligned to the contigs using bowtie with default parameters to determine the coverage of each antimicrobial resistance (AMR) category (Langmead and Salzberg, 2012). The coverage of an AMR category was calculated using the following formula:


$$\text{Coverage}=\sum_{1}^{n}\left(\frac{N\times 10^{9}}{L\times S}\right)$$

Here, *N* represents the number of reads mapped to ARG ORFs, *L* is the length of the target ARG ORFs sequence, *n* is the number of ARG ORFs within the specific AMR category, and *S* is the total number of reads in the respective sample (Ma et al., 2016).

To analyze the statistical comparisons, the Mann–Whitney test was employed. The Benjamini–Hochberg method was used for post-hoc testing to control the false discovery rate (FDR). We also utilized ordinal logistic regression analysis with SPSS 23.0 to assess potential significant differences in the total coverage and Shannon diversity index of the AMR category across the different groups.

## 3. Results

### 3.1. Analysis of the microbial composition

**Figure 1:**
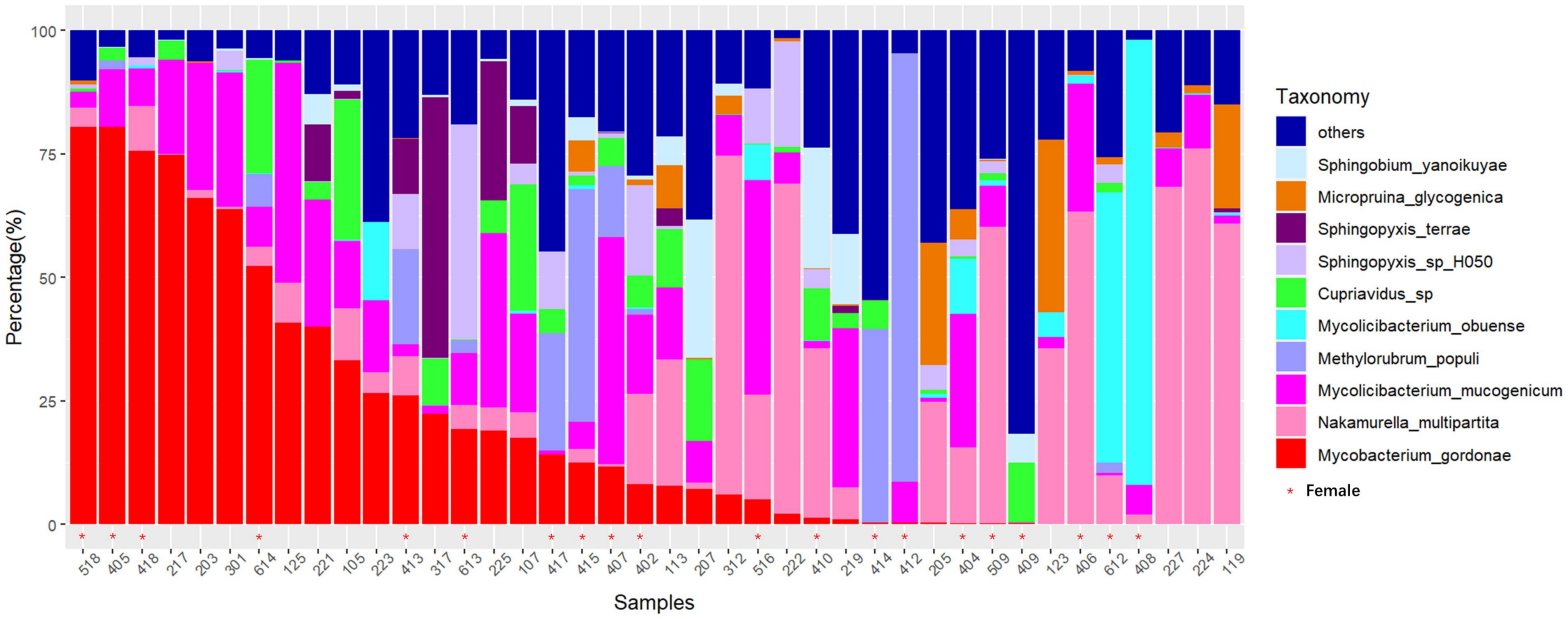
Stacked bar chart of the relative abundance of microbial species in drain pipes at the species level (sorted by *M. gordonae*). The *x*-axis represents the dormitory ID.

In this study, the microbial composition of drain pipes was analyzed using metagenomic sequencing. For two duplicate samples, we observed a strong correlation between the samples (both with Pearson correlation coefficient >0.999 and *p* < 2.2e^−16^), suggesting minimal interference from the sequencing libraries (Supplementary Figure S1). One of these duplicate samples was randomly selected for further analysis. There were 12 phyla present, with Proteobacteria and Actinobacteria being the dominant phyla. A total of 147 genera were annotated, with *Mycobacterium* and *Nakamurella* being the dominant genera. At the species level, *Mycobacterium gordonae* and *Nakamurella multipartita* were the most abundant in several dormitory samples. In fact, *M. gordonae* reached a relative abundance as high as 75% in certain dormitories (Figure 1). At the genus level, *Mycobacterium* and *Mycolicibacterium* were the most abundant (Supplementary Figure S2).

### 3.2. Analysis of microbial influencing factors

This study investigated the factors influencing the microbial community structure within dormitory drain pipes. The stress levels for Bray–Curtis dissimilarity and Euclidean distance in the NMDS analysis were 0.223 and 0.151, respectively. The envfit analysis revealed a weak yet significant association between gender and the microbial community composition, as indicated by both Bray–Curtis dissimilarity and Euclidean distance (Supplementary Figure S3, *R*^2^ = 0.3, *p* < 0.005). The Anosim analysis revealed that multiple factors had a significant influence on the microbial community structure in the drain pipes. Gender (*R* = 0.186, *p* = 0.001), the number of residents (*R* = 0.186, *p* = 0.001), and the hometowns of the dormitory residents (*R* = 0.134, *p* = 0.006) were found to have a significant impact. While the use of hand sanitizer did not show a significant effect on the microbial community structure (*R* = −0.069, *p* = 0.776), using hand sanitizer four or more times per week had a significant impact on the structure (*R* = 0.193, *p* = 0.031). The results of the multivariate ordered logistic regression analysis also indicate a significant difference in the Shannon index between the gender groups (Table 1, *p* = 0.002).

**Table 1:** Ordinal logistic regression analysis of alpha diversity indices of the microbiome community among different groups.

	Shannon		Simpson		Chao1	
	*p*-value	OR	*p*-value	OR	*p*-value	OR
Gender	0.002	0.014	0.268	0.562	0.920	1.066
Distance of hometown	0.139	0.373	0.368	0.482	0.047	0.258
Number of residents	0.787	0.839	0.960	0.969	0.192	0.421
Frequency of hand sanitizer usage	0.548	1.548	0.814	1.186	0.568	1.520
Antibiotic drugs usage	0.033	10.805	0.348	8.109	0.057	8.109
Occurrence of colds	0.198	0.368	0.057	0.484	0.353	0.486

**Figure 2:**
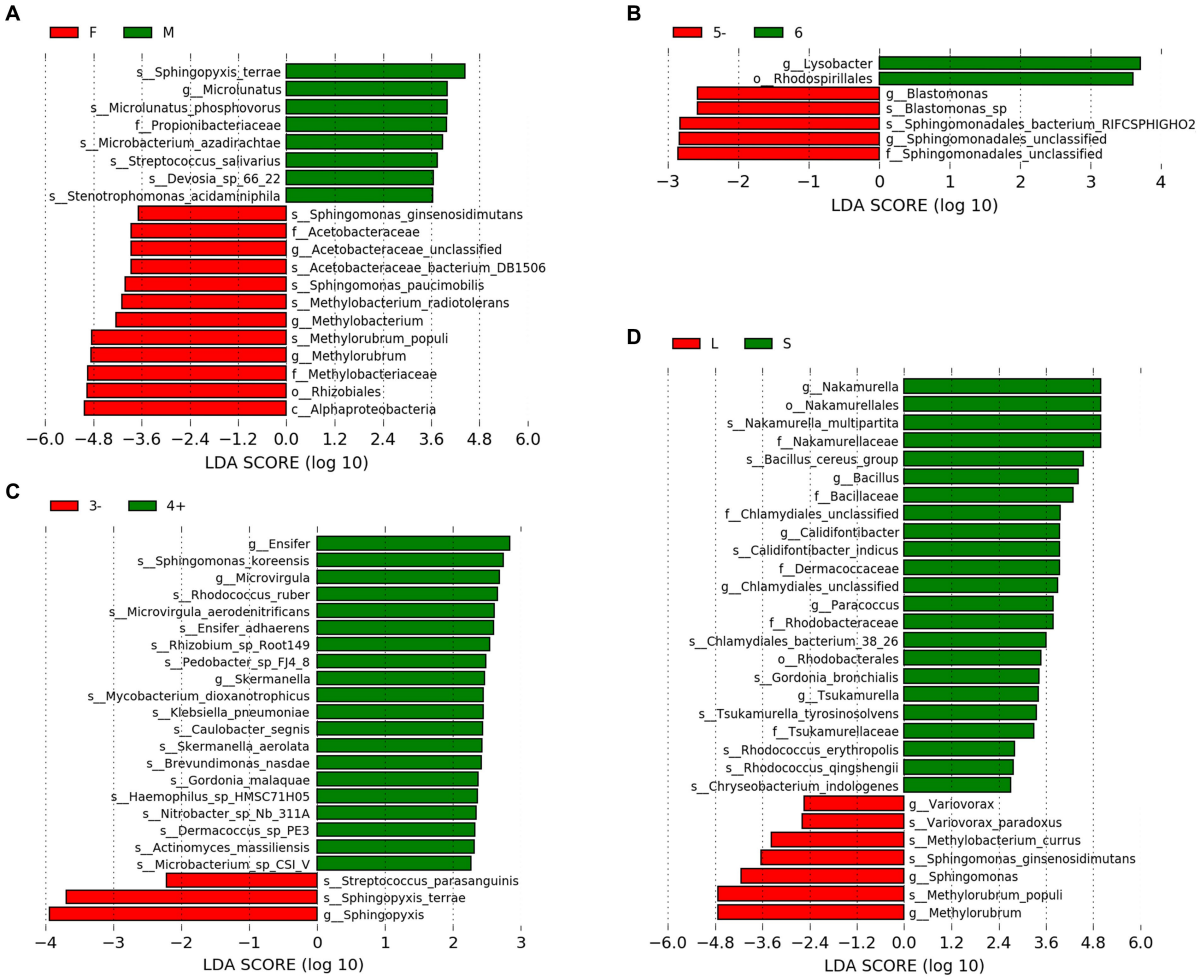
LEfSe results. **(A)** Gener. **(B)** Number of residents. **(C)** Frequency of hand sanitizer usage. **(D)** Distance of hometowns. “F” and “M” indicate the female and male groups, respectively. “5−” represents the group with residents equal to or less than 5, which “6” signifies the group with exactly 6 residents. “3−” means the group uses hand sanitizer three times or fewer per week, while “4+” means the group uses hand sanitizer four or more times per week. “L” and “S” indicate the group from closer and distant hometowns, respectively.

LEfSe analysis was employed to identify species exhibiting significant differences between groups (Figures 2A–D). With regard to gender, the male group demonstrated a significant increase in the relative abundance of six species, including *Microlunatus phosphovorus*, whereas the female group exhibited a significant increase in the relative abundance of five species, such as *Sphingomonas paucimobilis* and *Methylobacterium radiotolerans*. Concerning the number of residents, dormitories housing five or fewer individuals (5−) showed a significant increase in the relative abundance of two species. In terms of hand sanitizer usage, the group that frequently used hand sanitizer displayed a significant increase in the relative abundance of 17 species, including *Sphingomonas koreensis*, whereas the group that used it less frequently exhibited a significant increase in the relative abundance of two species, including *Streptococcus parasanguinis*. Regarding the hometowns of dormitory residents, the group from closer hometowns demonstrated a significant increase in the relative abundance of nine species, including *Nakamurella multipartita*, while the group from distant hometowns showed a significant increase in the relative abundance of four species, such as Var*iovorax paradoxus*.

The presence of a cold amongst dormitory members (*R* = −0.158, *p* = 0.963) do not influence the microbial structure of the dormitory drain pipes. The ordered logistic regression analysis findings align with those of Anosim. The Shannon index for whether dormitory members take antibiotics differs in the ordered logistic regression analysis (*p* = 0.03). However, it should be noted that this difference is not consistent across all analysis methods and the group homogeneity is poor (3 dormitories taking antibiotics and 37 not).

### 3.3. Analysis of ARGs, toxins, and virulence factors

**Figure 3:**
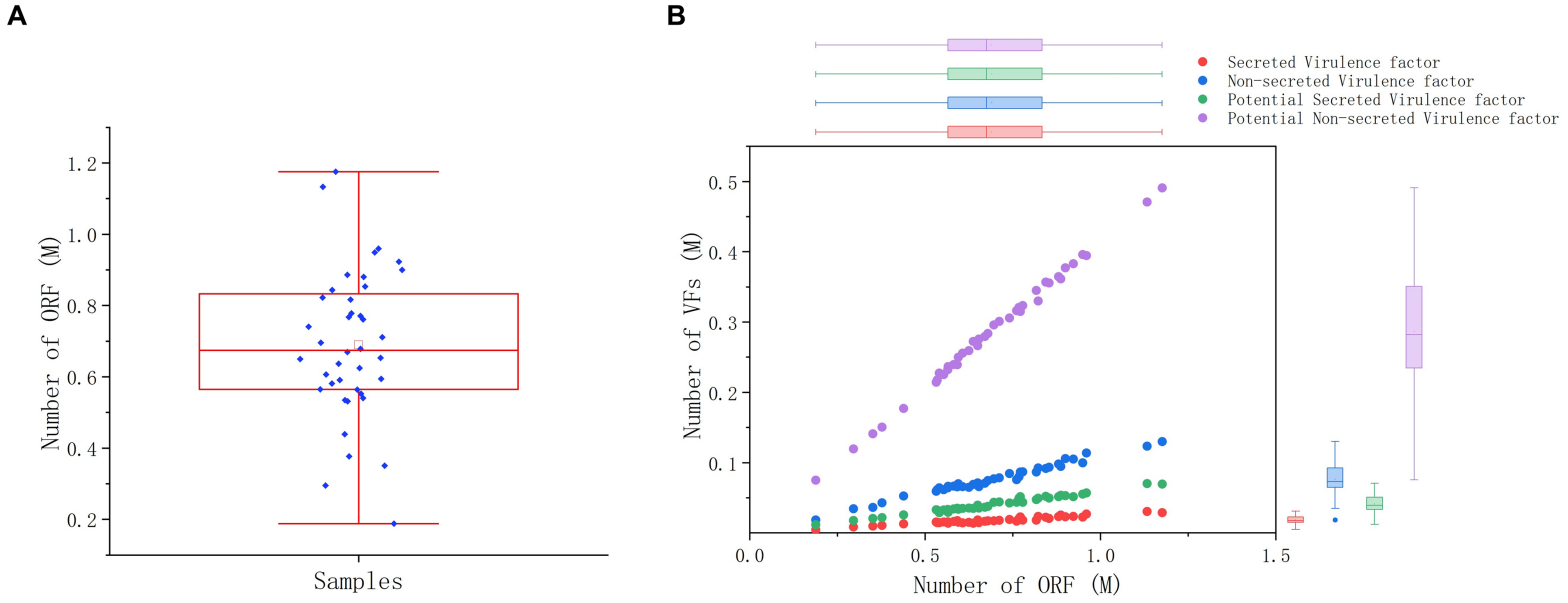
ORF and virulence factor analysis of microbial species in drain pipes. **(A)** Box plot of ORF number. **(B)** Distribution of ORF and virulence factors.

The PathoFact tool was used to analyze the virulence factors, toxins, and ARGs encoded by the microbiota in 40 dormitory samples. The number of predicted open reading frames (ORFs) in the dormitory samples averaged at 689,835.5, with a range from 187,852 to 1,175,647 (Figure 3A). Approximately 0.08% of ORFs encoded ARGs, encompassing a total of 30 different types. The most predominant type of AMR encoding was multidrug, constituting approximately 30.3% (Supplementary Figure S4). In contrast to the observed differences in microbial composition among samples, the distribution of resistance genes exhibited significant similarity. Most of these ARGs observed in the samples (mean = 60.82, StDev = 7.93) could not be attributed to a specific host genus (Supplementary Table S1), suggesting that the antibiotic resistance observed in the samples may originate from multiple sources or organisms. We also did not detect any significant differences in the coverage of these ARG types between different genders after performing the Benjamini–Hochberg process. We observed a significant increase in the total coverage of ARGs among the group of members who took antibiotics (*p* = 0.033), indicating a higher overall amount of ARGs (Table 2). However, when comparing the Shannon diversity index of AMR categories across different groups, we did not find a significant difference. This suggests that the composition of AMR categories does not vary significantly between these groups. Of the ORFs identified, 2.66% encoded secretory virulence factors, 11.11% encoded non-secretory virulence factors, 5.94% encoded potential secretory virulence factors, and 41.53% encoded potential non-secretory virulence factors. The number of ORFs encoding various types of virulence factors was strongly correlated with the total number of ORFs across diverse samples (Figure 3B), indicating a relatively conserved gene composition of the microbiota in dormitory washbasin drain pipes.

**Table 2:** Ordinal logistic regression analysis of the total coverage and Shannon diversity index of the AMR category among different groups.

	Total coverage		Shannon index	
	*p*-value	OR	*p*-value	OR
Gender	0.780	1.194	0.350	0.544
Distance of hometown	0.531	0.664	0.951	0.961
Number of residents	0.828	1.151	0.727	0.795
Frequency of hand sanitizer usage	0.956	1.041	0.415	1.831
Antibiotic drugs usage	0.038	10.299	0.899	1.146
Occurrence of colds	0.947	0.950	0.423	0.534

Approximately 1.25% of ORFs in the genome sequences of the sampled bacterial strains encoded pathogenic toxins, with 0.21% of ORFs encoding secreted toxins, including 50 different toxin types, while 1.04% of ORFs encoded non-secreted toxins, including 196 different toxin types. The main families of secreted toxins included Acr B/Acr D/Acr F family, Glyoxalase/Bleomycin resistance protein/Dioxygenase superfamily, and Cupin domain. Non-secretory toxin types mainly included outer membrane proteins, phosphotransferase enzyme family, putative hemolysin, nitroreductase family, and others. Additionally, a small number of YlaH-like protein, YlbE-like protein, and YebO-like protein were present in some dormitory samples.

The analysis also revealed that about 0.03% of ORFs in the genome sequences encoded antibiotic resistance genes, including 30 types, with an average of 25 antibiotic genes per dormitory sample strain. The most common type of encoded antimicrobial resistance was multidrug resistance, accounting for 30.3% of all antibiotic resistance genes. Multidrug-resistant bacteria can cause complex and difficult-to-treat infections, as they are resistant to three or more types of antibacterial drugs commonly used in clinics.

## 4. Discussion

At present, there have been extensive studies conducted on microbial pollution sources such as toilet aerosols and showerheads. The toilet, being a site where aerosols are easily generated and spread, emerges as the primary source of aerosols within the bathroom (Li et al., 2020). The forceful water flow generated during flushing can propel around 40 to 60% of particles above the toilet seat, leading to their wide dispersion. These particles can reach heights of up to 106.5 cm from the ground, potentially exposing individuals indoors to viruses present in the water mist. Moreover, showerheads have been found to harbor substantial quantities of bacteria. Research has consistently demonstrated the presence of abundant mycobacterial communities in showerheads within American and European households, whereas this phenomenon is not observed to the same extent in Japan, implying regional variations in bacterial habitat preferences (Ichijo et al., 2014; Gebert et al., 2018). While investigations into toilet drain pipes have predominantly focused on hospital environments or domestic water systems, it is noteworthy that sink drain pipes are susceptible to colonization by multiple drug-resistant bacteria (Kotay et al., 2017). For example, sink drain pipes have been implicated in the transmission of multidrug-resistant *Klebsiella pneumoniae* infections (Buchan et al., 2019). Hence, there exists a critical need for comprehensive research on pathogenic microorganisms present in the drain pipes of dormitory washbasins.

The microbial community in the drain pipe of dormitory washbasins contains various types of pathogenic bacteria. The variation in the abundance of *M. gordonae* observed among the samples in Figure 1 illustrates the intricate nature of the microbial community in the drain pipes, while the presence of numerous other taxa beyond the top 10 abundances signifies additional microbial diversity. Although *M. gordonae* is the most common species found in most dormitory samples, it is considered the least pathogenic of all mycobacteria. However, studies have reported that it can cause clinically significant diseases in immunocompetent individuals, including lung infections in young patients with normal immune function. LEfSe analysis showed that *M. phosphovorus* is associated with sick building symptoms (SBS) among gender factors (Fu et al., 2021). *Sphingomonas paucimobilis* can infect people with low immunity, and *M. radiotolerans* is linked to breast cancer (Xuan et al., 2014; Alkhatib et al., 2022). *Blastomonas* sp. can cause chronic kidney disease in population factors, while *Bacillus cereus group*, commonly known as the pathogen of food poisoning, can also cause local wound infection and systemic diseases in the grouping of hometown factors (Ehling-Schulz et al., 2019).

In the context of hand sanitizer usage frequency, it has been found that the group of individuals who use hand sanitizer frequently tend to have a higher presence of pathogenic bacteria. For instance, *Microvirgula* has been linked to thyroid cancer induction, *S. koreensis* is resistant to drugs, and the accumulation of bacteria in the facility pipeline system has been associated with pollution (Johnson et al., 2018). Hand sanitizer, acting as a stress factor, causes a surge in the discharge of antibacterial components into the water environment. As a result, the survival advantage of resistant strains in drain pipes becomes more evident, potentially accelerating the spread of microbial tolerance genes in the environment and leading to the emergence of more pathogenic bacteria. Therefore, further research is needed to determine how hand sanitizer can be used more effectively to prevent the aforementioned situation.

The analysis of virulence factors in the genome sequences of 40 dormitory samples revealed that there were more types and quantities of non-secretory toxins than secretory toxins. However, secretory toxins may play a more direct role in the pathogenic process. Among the secreted toxins, the mutation of Acr B/Acr D/Acr F family genes affects the antibiotic resistance of *Escherichia coli* (Kobylka et al., 2020), while the bleomycin resistance protein enables the tolerance to bleomycin. Regarding non-secretory toxins, outer membrane protein is a crucial material basis of bacterial pathogenicity and can inhibit the host immune response (Rollauer et al., 2015). Bacterial hemolysin, on the other hand, can cause inflammatory reaction via membrane injury and cell lysis, thereby playing a pathogenic role (Goebel et al., 1988). Additionally, some toxins, such as YlaH-like protein and YlbE-like protein, only appeared in individual dormitory samples and their effects are unknown. Therefore, further research is necessary to investigate the molecular mechanisms of virulence factors, toxin genes, and their relationship with the influencing factors of dormitory flora structure.

This study revealed that there are various types of antimicrobial resistance (AMR) in gender grouping, including multidrug, tetracycline, aminoglycoside, beta-lactam, MLS, peptide, and glycopeptide. The widespread use of tetracycline antibiotics has led to the extensive presence of tetracycline resistance genes in gram-negative bacteria, posing a severe threat to human and animal health (Zheng et al., 2022). MLS (macrolides-lincosamides-Streptogramins) is a group of antibiotics that share a similar antibacterial spectrum and mechanism, including 14, 15, and 16-membered macrolides, lincomycin, and streptomycin. Glycopeptide antibacterial agents are a group of natural and semi-synthetic glycosylated peptides that exhibit antibacterial activity against gram-positive organisms by inhibiting cell wall synthesis (Zeng et al., 2016).

## 5. Conclusion

In summary, the factors influencing the microbial community structure of the dormitory washbasin drain pipe are complex. The investigated factors have a slight influence on the drain pipe microbial community, with gender exerting a discernible influence. In contrast to the observed differences in microbial composition among samples, the distribution of resistance genes shows relatively small changes among samples. The presence of numerous pathogenic bacteria in the drain pipe carrying a large number of antibiotic resistance genes and virulence factors can cause indoor environmental contamination and increase the potential risk of disease transmission. Antibiotics may be a contributing factor in the overall increase of ARGs. It is important to note that further studies with a larger sample size are required to establish a more robust conclusion. Therefore, relevant authorities should enhance health monitoring, be vigilant about microbial pollution of dormitory drain pipes, and ensure the health and safety of residents.

## Data availability statement

The datasets presented in this study can be found in online repositories. The names of the repository/repositories and accession number(s) can be found in the article/Supplementary material.

## Ethics statement

The manuscript presents research on animals that do not require ethical approval for their study.

## Author contributions

YH: Conceptualization, Data curation, Formal analysis, Investigation, Methodology, Writing – original draft. KZ: Conceptualization, Data curation, Formal analysis, Investigation, Methodology, Writing – original draft. NL: Conceptualization, Data curation, Writing – original draft. SW: Conceptualization, Funding acquisition, Methodology, Supervision, Writing – review & editing.

## Footnotes

^1^ https://huttenhower.sph.harvard.edu/kneaddata/
