# Supplementary material for: Composition, antibiotic resistance, and virulence analysis of microbiota in dormitory drain pipes
Source: Front Microbiol. 2023 Nov 13;14:1272605. doi: 10.3389/fmicb.2023.1272605 (PMC10679431; doi:10.3389/fmicb.2023.1272605)
Supplement: Supplementary file 1 [file Data_Sheet_1.DOCX]

Supplementary Material

# Supplementary Figures and Tables

## Supplementary Figures

**Supplementary Figure S1.** Stacked bar chart of the relative abundance of microbial species in two duplicated samples at the species level. The x-axis represents the dormitory ID.

**Supplementary Figure S2.** Stacked bar chart of the relative abundance of microbial species in drain pipes at the genus level (sorted by *Mycobacterium*). The x-axis represents the dormitory ID.

**Supplementary Figure S3.** Nonmetric multidimensional scaling (NMDS) plot displaying the taxonomic similarity of the microbial community in dormitory drain pipes. The stress levels are indicated at the top. The arrows on the NMDS plot represent the envfit correlations of the microbial community composition with influencing factors. (A) Bray-Curtis dissimilarity. (B) Euclidean distance.

**Supplementary Figure S4.** Stacked bar chart illustrating the distribution of various AMR categories in drain pipes. The x-axis represents the dormitory ID.

## Supplementary Tables

**Supplementary Table S1.** Top 3 genus carrying antibiotic resistance genes in each sample (%).

| Samples | Genus 1 | Genus 2 | Genus 3 |
| --- | --- | --- | --- |
| 105 | *Mycobacterium* (6.16) | *Zoogloea* (2.89) | *Cupriavidus* (1.88) |
| 107 | *Pseudomonas* (7.39) | *Mycobacterium* (6.57) | *Stenotrophomonas* (6.57) |
| 113 | *Stenotrophomonas* (7.01) | *Mycobacterium* (6.48) | *Sphingobium* (2.80) |
| 119 | *Mesorhizobium* (2.27) | *Nakamurella* (2.01) | *Pseudoxanthomonas* (1.74) |
| 123 | *Nakamurella* (3.31) | *Mesorhizobium* (1.98) | *Phenylobacterium* (1.65) |
| 125 | *Mycobacterium* (12.61) | *Sphingopyxis* (1.85) | *Pseudonocardia* (1.68) |
| 203 | *Mycobacterium* (7.39) | *Bradyrhizobium* (3.29) | *Reyranella* (2.46) |
| 205 | *Paracoccus* (2.79) | *Gordonia* (2.61) | *Rhodococcus* (2.42) |
| 207 | *Stenotrophomonas* (8.48) | *Acinetobacter* (6.72) | *Mycobacterium* (4.00) |
| 217 | *Mycobacterium* (14.01) | *Methylobacterium* (1.91) | *Sphingopyxis* (1.49) |
| 219 | *Pseudomonas* (6.82) | *Stenotrophomonas* (4.90) | *Acinetobacter* (3.47) |
| 221 | *Mycobacterium* (8.33) | *Cupriavidus* (4.39) | *Stenotrophomonas* (3.79) |
| 222 | *Nakamurella* (3.05) | *Mycobacterium* (2.89) | *Sphingopyxis* (2.41) |
| 223 | *Mycobacterium* (5.39) | *Stenotrophomonas* (5.21) | *Gordonia* (5.03) |
| 224 | *Nakamurella* (2.70) | *Gordonia* (1.84) | *Sphingopyxis* (0.86) |
| 225 | *Mycobacterium* (7.34) | *Sphingopyxis* (2.18) | Exophiala (1.98) |
| 227 | *Mesorhizobium* (3.75) | *Acinetobacter* (2.46) | *Nakamurella* (2.33) |
| 301 | *Mycobacterium* (9.66) | *Bradyrhizobium* (4.43) | *Methylobacterium* (3.42) |
| 312 | *Mycobacterium* (5.56) | *Pseudomonas* (4.40) | *Nakamurella* (3.01) |
| 317 | *Mycobacterium* (10.17) | *Stenotrophomonas* (5.73) | *Acidovorax* (3.14) |
| 402 | *Mycobacterium* (5.50) | *Acinetobacter* (3.98) | *Acidovorax* (3.16) |
| 404 | *Micromonospora* (3.15) | *Mycolicibacterium* (2.60) | *Methylobacterium* (2.60) |
| 405 | *Mycobacterium* (9.65) | *Methylobacterium* (5.70) | *Cupriavidus* (2.41) |
| 406 | *Nakamurella* (4.33) | *Methylobacterium* (1.68) | *Bosea* (1.40) |
| 407 | *Methylobacterium* (5.20) | *Mycobacterium* (5.20) | Novo*Sphingobium* (2.60) |
| 408 | *Mycolicibacterium* (2.74) | *Mesorhizobium* (2.40) | *Phenylobacterium* (2.05) |
| 409 | *Methylobacterium* (3.36) | *Pleomorphomonas* (3.07) | *Stenotrophomonas* (2.78) |
| 410 | *Stenotrophomonas* (5.59) | *Pseudomonas* (4.83) | *Acinetobacter* (4.68) |
| 412 | *Methylobacterium* (16.49) | *Sphingomonas* (5.85) | *Stenotrophomonas* (5.32) |
| 413 | *Mycobacterium* (13.08) | *Methylobacterium* (4.62) | *Stenotrophomonas* (4.42) |
| 414 | *Acinetobacter* (7.55) | *Methylobacterium* (7.34) | *Pseudomonas* (7.34) |
| 415 | *Mycobacterium* (6.12) | *Stenotrophomonas* (3.96) | *Sphingomonas* (3.96) |
| 417 | *Mycobacterium* (7.69) | *Stenotrophomonas* (6.27) | *Methylobacterium* (5.41) |
| 418 | *Mycobacterium* (12.50) | *Methylobacterium* (11.06) | *Sphingomonas* (3.12) |
| 509 | *Stenotrophomonas* (3.60) | *Nakamurella* (3.06) | *Rhodococcus* (1.80) |
| 516 | *Mycobacterium* (9.52) | *Methylobacterium* (8.49) | *Nakamurella* (2.28) |
| 518 | *Mycobacterium* (6.51) | *Acidovorax* (3.02) | *Bradyrhizobium* (2.79) |
| 612 | *Stenotrophomonas* (6.30) | *Methylobacterium* (4.04) | *Micromonospora* (3.39) |
| 613 | *Acinetobacter* (6.15) | *Mycobacterium* (5.32) | *Stenotrophomonas* (3.16) |
| 614 | *Mycobacterium* (7.01) | *Methylobacterium* (5.84) | *Caulobacter* (3.21) |
