# Supplementary figures and images for: Composition, antibiotic resistance, and virulence analysis of microbiota in dormitory drain pipes
Source: Front Microbiol. 2023 Nov 13;14:1272605. doi: 10.3389/fmicb.2023.1272605 (PMC10679431; doi:10.3389/fmicb.2023.1272605)

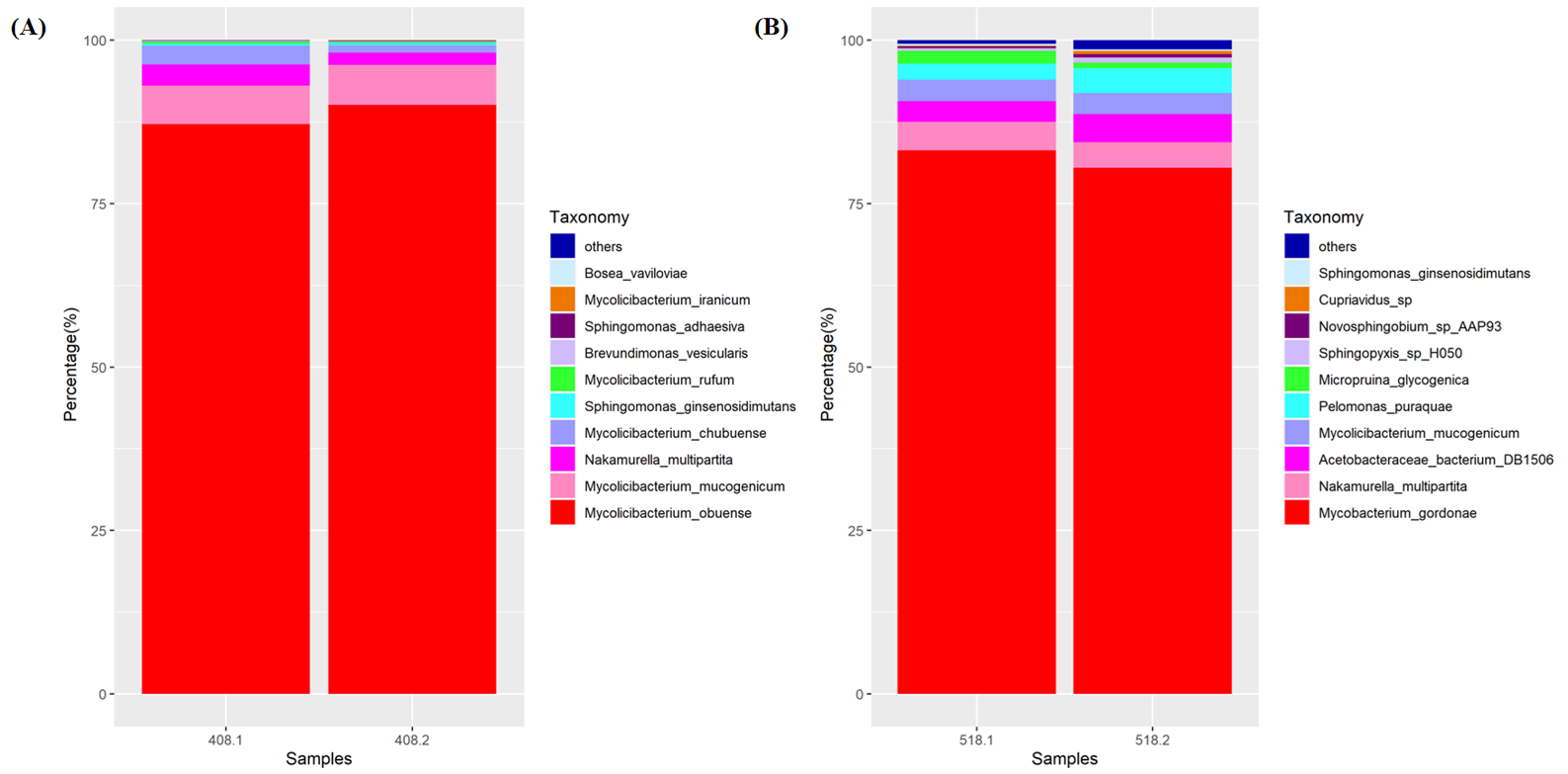

Supplement: Supplementary file 2 [file Image_1.TIF]

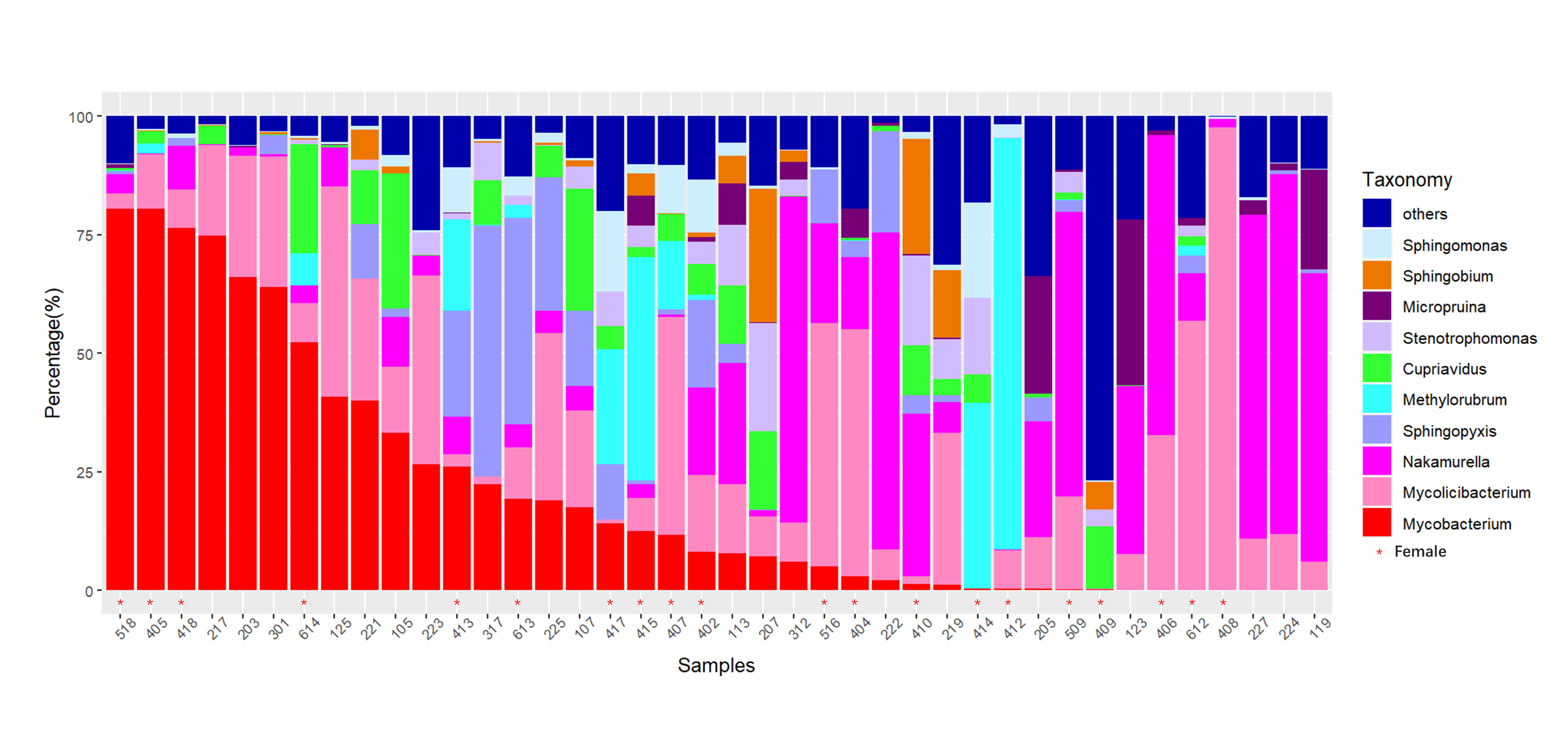

Supplement: Supplementary file 3 [file Image_2.TIF]

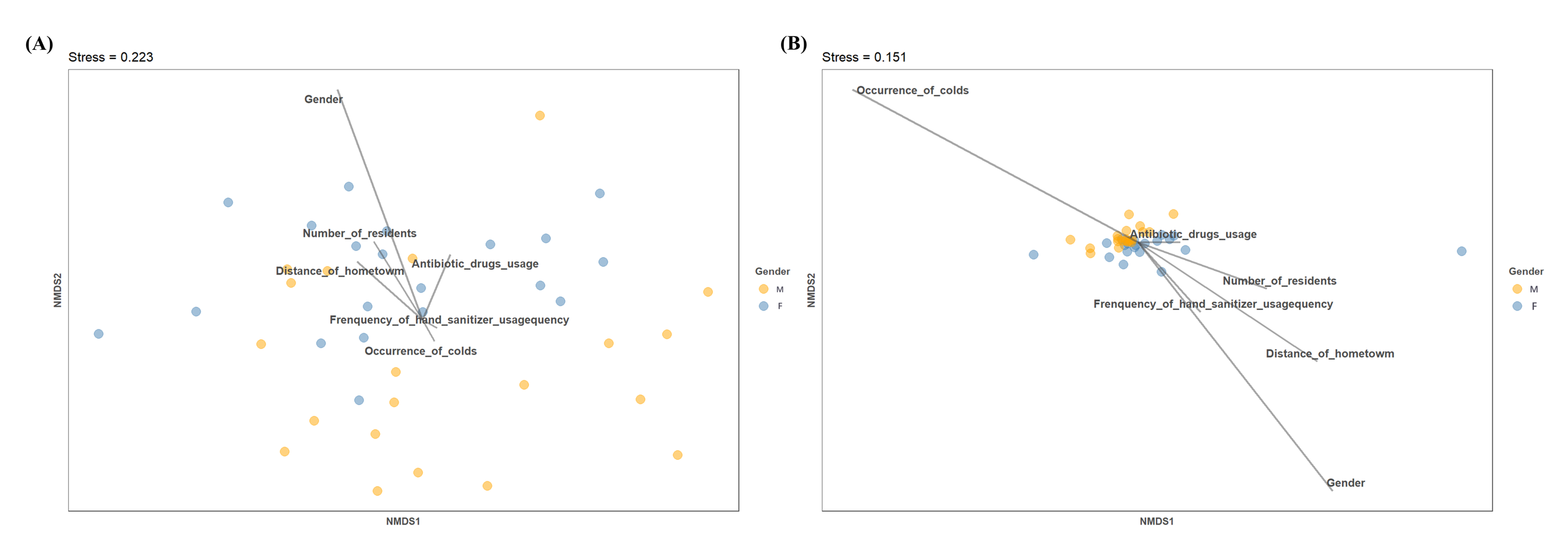

Supplement: Supplementary file 4 [file Image_3.TIF]

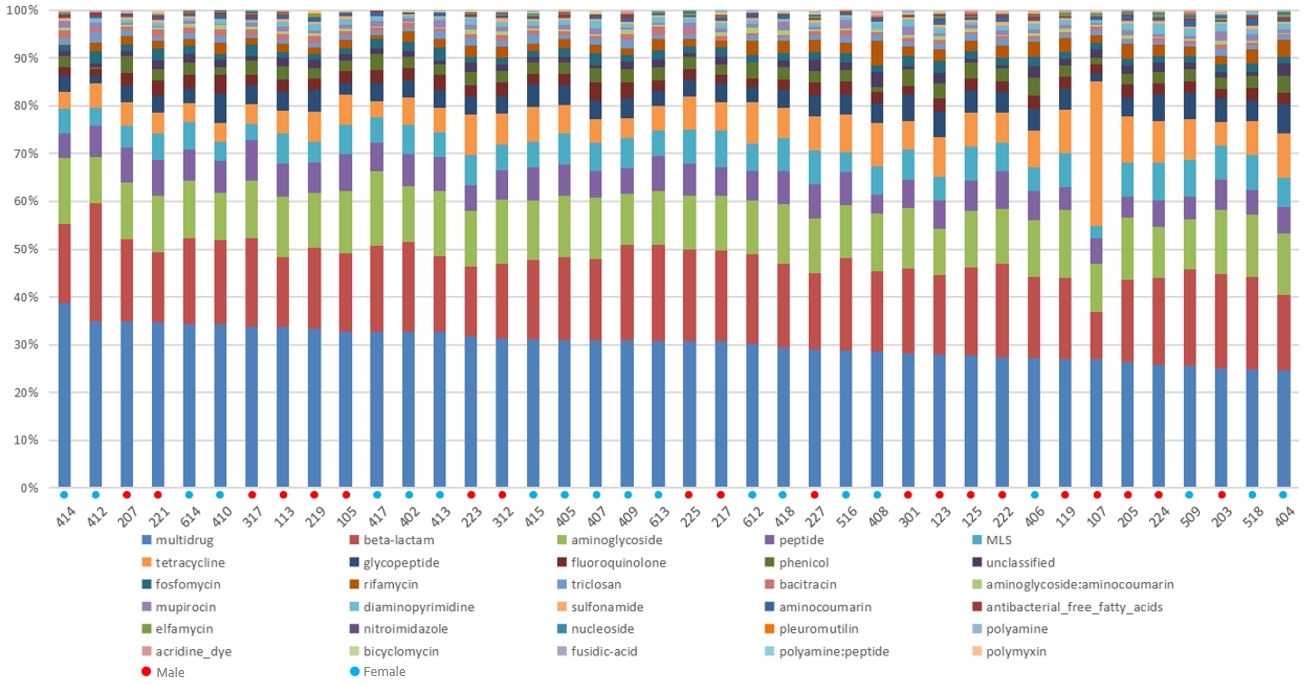

Supplement: Supplementary file 5 [file Image_4.TIF]
